# Validation of CD4^+^ T-cell and viral load data from the HIV-Brazil Cohort Study using secondary system data

**DOI:** 10.1186/s12879-018-3536-4

**Published:** 2018-12-04

**Authors:** Alex Jones Flores Cassenote, Alexandre Grangeiro, Maria Mercedes Escuder, Jair Minoro Abe, Aluísio Augusto Cotrim Segurado

**Affiliations:** 1 Postgraduate Program in Infectious and Parasitic Diseases, University of São Paulo School of Medicine, São Paulo, Brazil; 2 Department of Preventive Medicine, University of São Paulo School of Medicine, São Paulo, Brazil; 3 São Paulo State Department of Health, Health Institute, São Paulo, Brazil; 4 Institute for Advanced Studies, University of São Paulo, São Paulo, Brazil; 5 Department of Infectious Diseases, University of São Paulo School of Medicine, São Paulo, SP Brazil

**Keywords:** HIV-Brazilian cohort study, TCD4+ − cell, Viral load, Validation, Medical records

## Abstract

**Background:**

The HIV-Brazil Cohort Study (HIV-BCS) is a research primarily based on data collection from medical records of people living with HIV/AIDS in Brazil. The aim of this study was to present the validating design and results for the laboratory biomarkers viral load and CD4+ T-cell count from the HIV-Brazil Cohort Study.

**Methods:**

A total of 8007 patients who were started cART from 2003 to 2013 were considered eligible for this study. Total follow-up time was 32,397 years. The median duration of follow-up was 3.51 years (interquartile range - IQR 1.63–6.13 years; maximum 11.51 years). We used secondary data from the Brazilian Laboratory Tests Control System (SISCEL). Incidence of lab testing rates per 100 person years (100 py) were used to compare the number of laboratory tests carried out among cohort sites considering different databases for CD4+ T-cell counts and HIV viral load assessments. Descriptive statistics including 95% confidence interval, Pearson correlation coefficient, Bland-Altman agreement analysis and kappa coefficient agreement were applied for analysis.

**Results:**

A total of 80,302 CD4+ T-cell counts and 79,997 HIV viral load assessments were observed in HIV-BCS versus 94,083 CD4+ T-cell counts and 84,810 viral loads from the Brazilian Laboratory Tests Control System. The general CD4+ T-cell HIV-BCS testing rate was 247 per 100 py versus 290 per 100 py and the viral load HIV-BCS testing rate was 246 per 100 py versus 261 per 100 py. The general correlation observed for the lowest quantitative CD4+ T-cell count before cART was 0.970 (*p* < 0.001) and for the log of the highest viral load before cART was 0.971 (*p* < 0.001). The general agreement coefficient for categorized CD4+ T-cell count was 0.932 (*p* < 0.001) and for viral load was 0.996 (*p* < 0.001).

**Conclusions:**

The current study confirms that biomarkers CD4^+^ T-cell count and viral load from the HIV-BCS have a high correlation and agreement with data from SISCEL, rendering both databases reliable and useful for epidemiological studies on HIV care in Brazil.

**Electronic supplementary material:**

The online version of this article (10.1186/s12879-018-3536-4) contains supplementary material, which is available to authorized users.

## Background

HIV/AIDS cohort studies based on clinic populations and medical records are becoming more abundant due in part to an increasing trend toward electronic medical records and advances in information technology [1]. In the absence or difficulty to obtain prospectively collected clinical data, the epidemiological studies of HIV infection often rely on a variety of secondary sources for patient information, including the patient report, medical records, and surveillance data [2].

Brazil is estimated to have 830,000 people living with HIV and, following national guidelines issued by the Ministry of Health, all should be treated with combined antiretroviral therapy (cART), provided free of charge through the public health sector. In this scenario, the HIV-Brazil Cohort Study (HIV-BCS) is being carried out as a nationwide research project, primarily based on data collection from medical records of people living with HIV/AIDS. Due to its long follow-up period and a significant number of observations, this study is recognized as an important asset to increase the availability of data on outcomes of the National AIDS Program related to the prescription of cART in public healthcare services [3].

In Brazil, AIDS epidemiological surveillance, apart from being based on information provided through the notification of cases recorded on the Notifiable Diseases Information System (Sistema de Informação de Agravos de Notificação - SINAN) and deaths recorded on the Mortality Information System (Sistema de Informação sobre Mortalidade - SIM), also draws information from two other systems: the Laboratory Tests Control System (Sistema de Controle de Exames Laboratoriais - SISCEL) and the Medication Logistics Control System (Sistema de Controle Logístico de Medicamentos - SICLOM). These databases comprise the basis of the National HIV/AIDS register in Brazil [4].

The SISCEL system has been developed with the aim of monitoring CD4 T lymphocyte (CD4^+^ T-cell) counts and HIV viral load (VL) assessments, biomarkers that are used to decide when patients should start treatment and to monitor patients already under antiretroviral therapy [4].

So far little effort has been made to correlate multiple sources of data [2]. Comparisons of medical records and HIV/AIDS surveillance data found good agreement for individual data [5–7]. A study performed in North Carolina tried to describe and quantify differences during the year of the first positive HIV test among patient reports, medical records, and HIV/AIDS surveillance data and concluded these measures could not reliably be used interchangeably as there was wide variability between them. Although the collection of data from patient reports or existing sources is convenient, cost-effective and efficient, there is significant variability among all sources [2]. Another recent research proposed to compare measures of retention in HIV care status based on both clinic visit data and HIV laboratory surveillance data. Although the authors have pointed out important limitations associated with definitions being used for retention, they concluded the combined use of laboratory and clinic visit-based data to measure retention in care provides a more accurate representation of the care status of HIV-infected patients than use of a single data source alone [8].

The assessment of the quality of data extracted from medical records is vital to correctly interpret the results obtained in the HIV-BCS and should help indicate critical points and open the door to further scientific production. The aim of this study was to present the validating design and results for the laboratory biomarkers CD4^+^ T-cell counts and HIV VL assessments from the HIV-BCS, using secondary data from SISCEL.

## Methods

### HIV-Brazil cohort study

HIV-BCS is an ambidirectional cohort involving 13 Brazilian sites, comprising 26 public health facilities in 11 cities across four of the five administrative regions of the country. Patients aged over 18 who were started on cART from 2003 to 2013 are enrolled in this cohort. The facilities were selected based on convenience, by region and city of location, availability of information on the clinical follow-up and the use of cART, and the existing infrastructure to conduct studies of this nature. The cities in which these facilities are located were chosen because they reflect the diversity of the epidemiological profile of AIDS in Brazil. Information about cohort sites, eligibility and inclusion criteria, data sources, outcomes, censoring criteria, availability of data and ethics statement have been previously reported [3].

The cohort data were obtained as part of the routine clinical care provided at the health services (routine-care-based cohort) and were abstracted from patients’ clinical records by trained abstractors onto standardized forms. These clinical records were reviewed at intervals not exceeding 6 months to investigate events recorded during the routine clinical follow-up visits performed within each period.

**Fig. 1:**
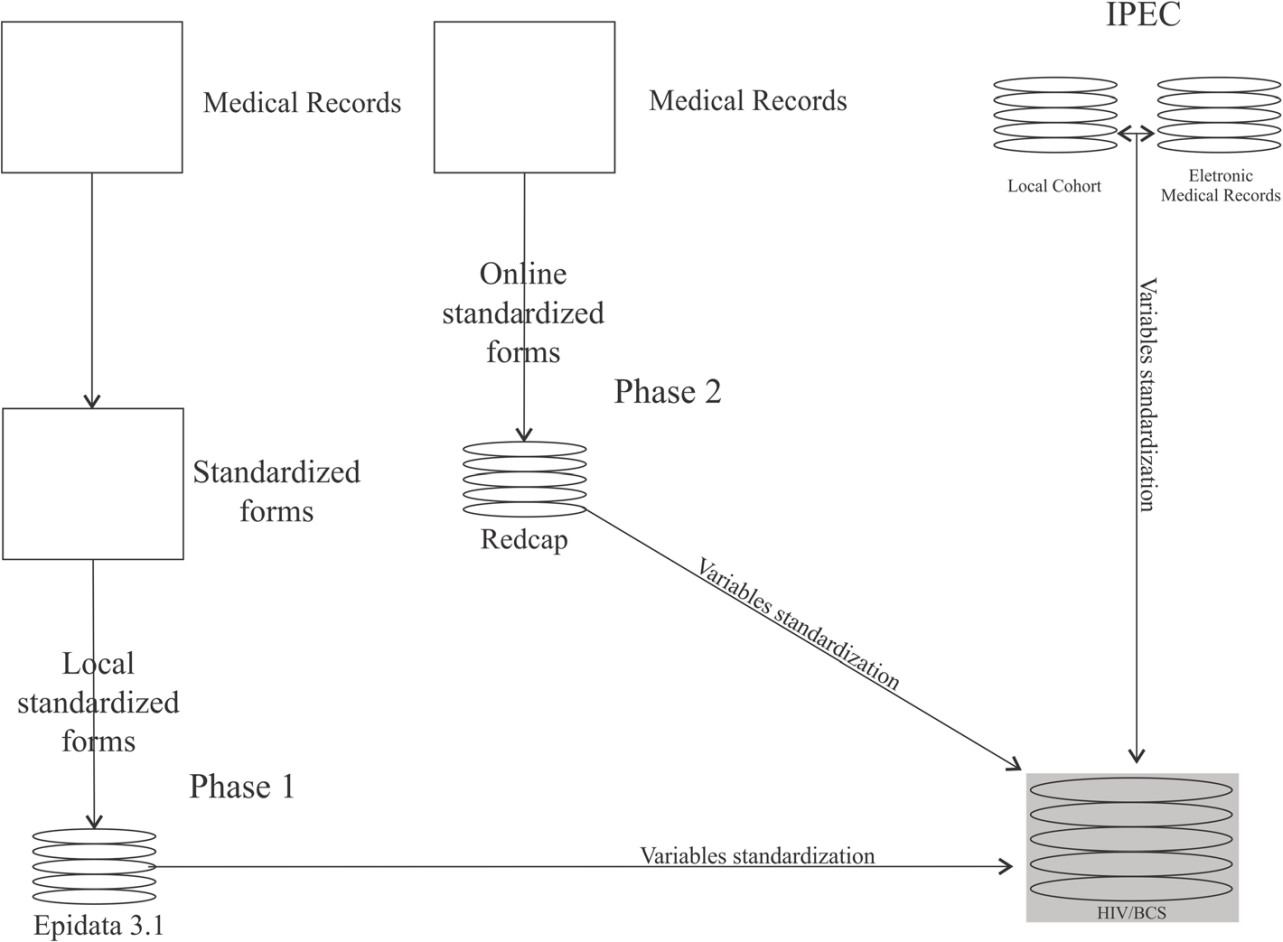
The structure of the HIV-BCS database with the different databases in different phases of the study (Phase 1 is retrospective and Phase 2 is prospective)

Different data collection strategies were used in the HIV-BCS cohort study: phase 1 –*retrospective cohort*– a standardized form was applied on patient medical reports and data entered onto a specific EpiData 3.1 form (The EpiData Association, Odense, Denmark); phase 2 – *prospective cohort* – an online standardized form was used and data entered directly from patients’ medical records into a REDCap (Research Electronic Data Capture, Nashville, United State) file; additionally, the IPEC site (retrospective and prospective) included data from their local cohort and electronic medical records after inclusion of all their patients who met the specific criteria in the study (Fig. 1).

For this evaluation, we used the following variables: patient code, historical laboratory results including CD4+ T-cell counts (number of cells per mm^3^) and VL assessments (absolute number of HIV RNA copies/ml and log of number of copies/ml) and date of blood sample collection.

### Brazilian laboratory test control system–SISCEL

Since 1997, the Department of Sexually Transmitted Disease and AIDS started to deploy the National Network of Laboratories (NNT) for carrying out T-cell counts (CD4^+^/CD8^+^) and HIV VL assessments. The main objective of the network is to monitor the course of HIV infection, guide the initiation of antiretroviral therapy and to provide immunological parameters for the prescription of chemoprophylaxis for opportunistic infections [4].

SISCEL was implemented in all Brazilian states in 2002, and its billing module enables laboratories affiliated to NNT to generate all the information required by the Ministry of Health for billing. Currently, SISCEL is being used in all Brazilian states, with 95 laboratories performing CD4+/CD8+ T-cells counts and 85 laboratories performing VL tests [4].

**Fig. 2:**
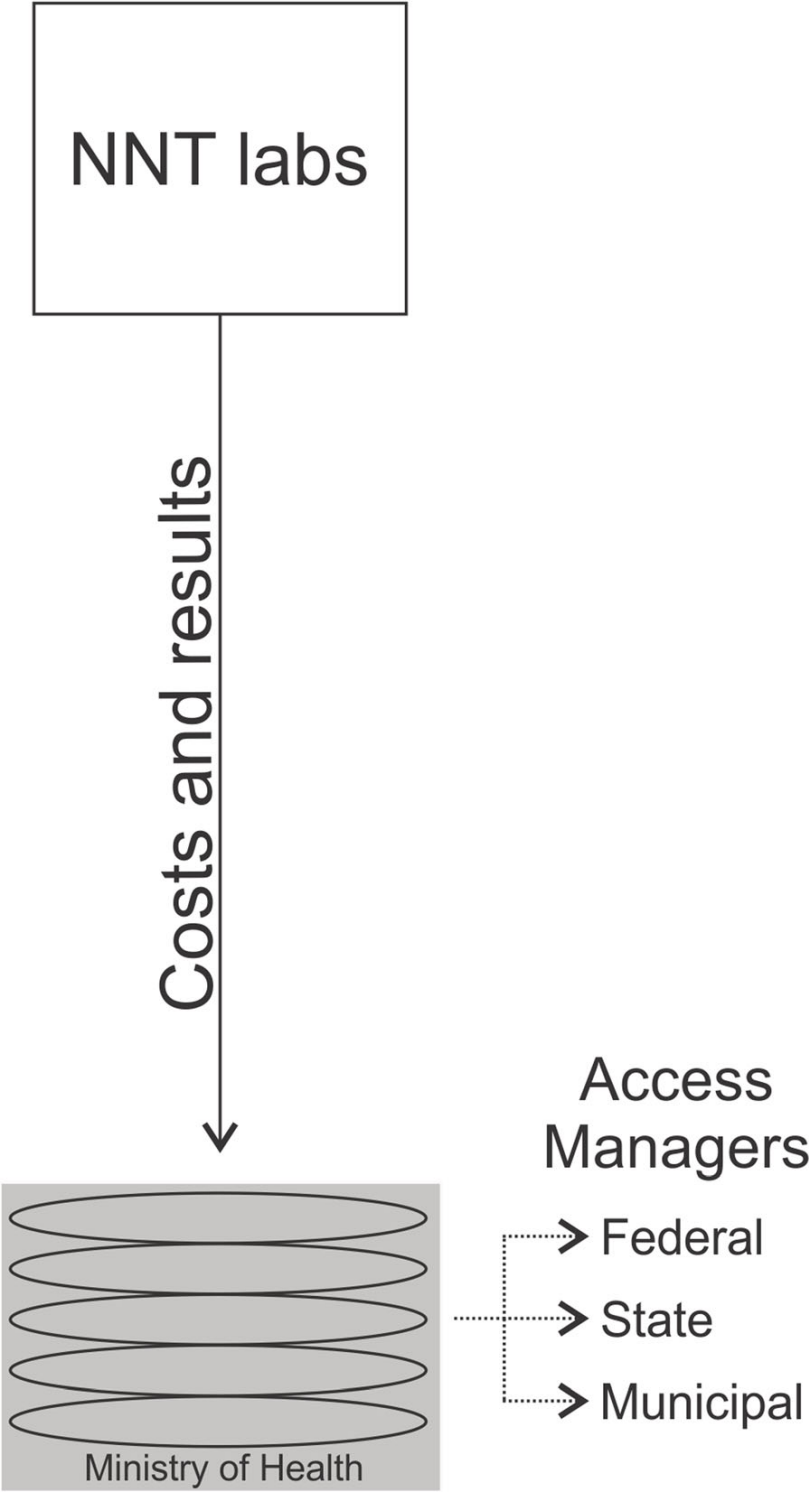
SISCEL operating system used by National Network of Laboratories (NNT) to feed the database with costs and laboratory results of T-cell counts (CD4+/CD8+) and HIV VL assessments

Information is fully stored in the central database in the Department of Sexual Transmitted Disease, AIDS and Viral Hepatitis of the Ministry of Health. This database is automatically fed by NNT laboratories and can be accessed by federal, state and municipal managers of STD and AIDS programs, using the Internet with data encryption (Fig. 2). There are two subsystems, one for VL assessments and another for CD4+/CD8+ T-cell counts. The SISCEL’s logical framework can be seen more detail in another study [5].

For this evaluation, we used following variables: patient code, date of birth, requesting institution, request date, sample collection date, result date, CD4^+^ T-cell and CD8^+^ T-cell count, total lymphocyte count, VL copy number and log, and qualitative result of VL (above or below assay lower limit).

### Matched system

A single code was generated for each patient in the different databases used in the HIV-BCS study. For patients enrolled in phase 1 and only updated during follow-up in phase 2, a specific field was created in the online REDcap form to enable entering the patient code used in the retrospective phase.

The concatenation between the HIV-BCS database and the National HIV/AIDS register database was a major challenge because the common identification variables in the two databases were: patient’s name, the patient’s mother’s name and date of birth. Thus, the bubble sort computer system was developed based on C# language to generate unique patient codes for cases that were 100% compatible. Additionally, a researcher evaluated the records that did not match to identify potential problems (for example, names incorrectly typed). The creation of this single registration enables a linkage with any database from the National HIV/AIDS register.

### Inclusion, censoring criteria and ethical aspects

**Fig. 3:**
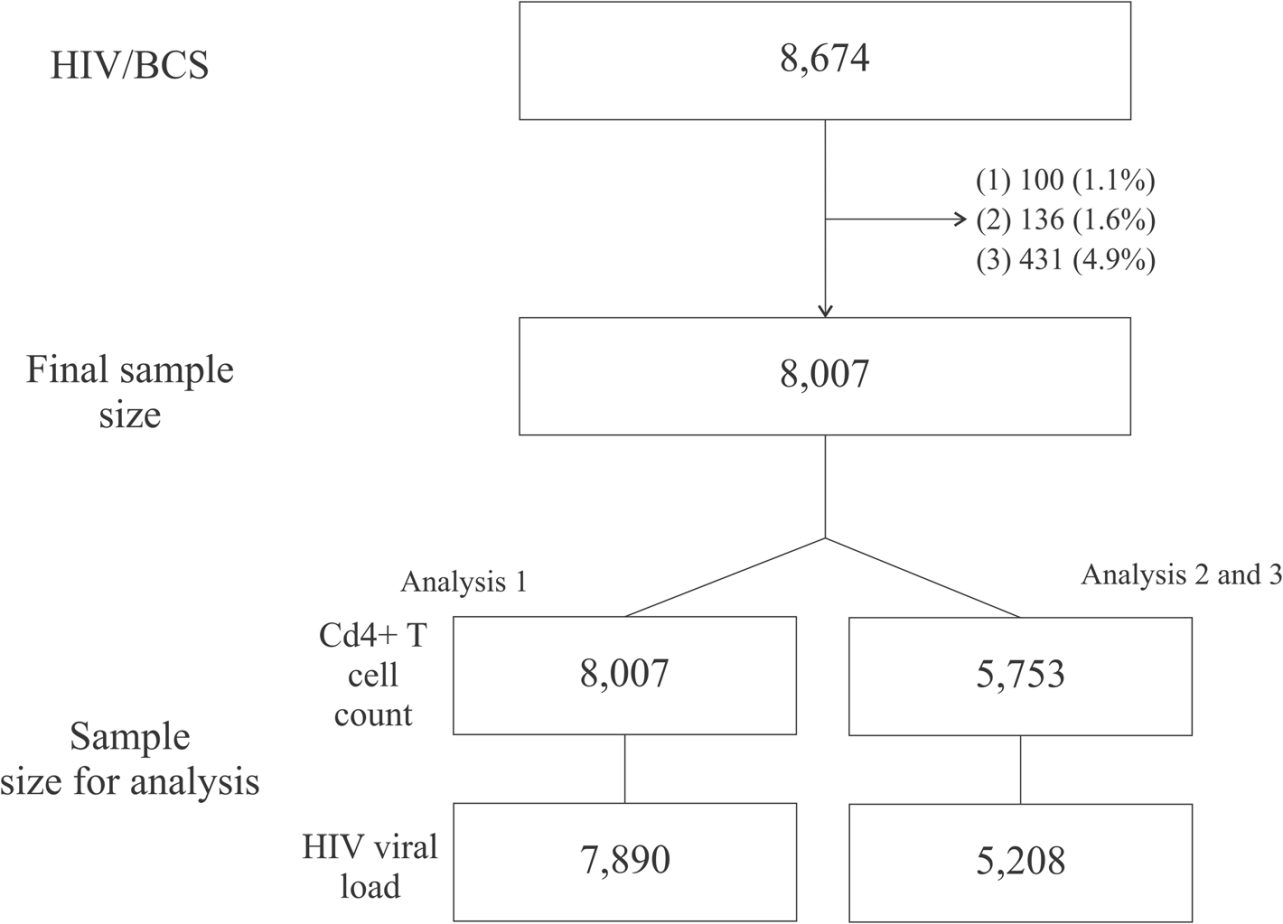
Final sample size flowchart. (1) no linkage code with National HIV/AIDS register surveillance; (2) no data available for CD4+ T-cell count or HIV viral load assessment during clinical follow-up; or (3) no data available for CD4+ T-cell count or viral load after initiating cART. Analysis 1 refers to descriptive statistics including mean, median, standard deviation (SD) and interquartile range (IQR) and proportions (%) for the ten first quantitative measures of CD4+ T-cell counts and categorized viral loads (above or below assay lower limit) after cART; analysis 2 including Pearson correlation coefficient and Bland-Altman agreement analysis for quantitative measures of lowest CD4+ T-cell count and log of highest viral load before cART; and analysis 3 is a Kappa coefficient agreement for categorized measures of lowest CD4+ T-cell count before cART and highest viral load before cART

For this specific paper we have adopted an inclusion criteria similar to the HIV-BCS [3] but some subjects were excluded (Figs. 1 and 3) no linkage code with National HIV/AIDS register surveillance (1.1% or 100 individual); (2) no data available for CD4^+^ T-cell count or HIV VL assessment during clinical follow-up (1.6% or 136 individuals); or (3) no data available for CD4+ T-cell count or VL after initiating cART (4.9% or 431 individuals).

The censoring date considered in the HIV-BCS was July 31, 2014 for patients that starting cART from January 01, 2003 to December 31, 2013. In addition, we used the dates of the last CD4^+^ T-cell counts and VL to censure SISCEL database and prevent entry of tests results beyond the date recorded in the cohort study.

In order to work with possible discrepancies between tests due to the lack of matching the exact tests dates (CD4+ T-cell or VL), the comparisons were made with another result of the same test considering the closest date, respecting an interval of 30 days.

HIV-BCS was approved by the Institutional Review Boards (IRB) of the participating sites: in the first phase, the IRB waived the requirement for written informed consent, given that confidentiality of the individual’s data was ensured at all stages of the project. In the second phase, all participants provided written consent for participation in the study. This specific study was approved by the Ethics Committee of the Medical School of the University of São Paulo (#229/13).

### Statistical analysis

The analysis used in this study aimed to summarize updated information from HIV-BCS and to show the validating process based on matching CD4^+^ T-cell counts and VL assessments between the HIV-BCS database and the SISCEL database. In the first approach (considering sample size showed Fig. 3 – Analysis 1), central tendency and dispersion statistics are used to characterize the cohort follow-up; incidence of lab testing rates per 100 person years (100 py) were used to compare the number of laboratory tests carried out among cohort sites considering different databases. In addition, proportion and 95% confidence intervals based on 1000 bootstrap samples [9] for qualitative lowest CD4+ T-cell before cART and higher VL before cART were studied. For the second approach three analysis strategies were used (Fig. 3 – Analysis 2 and 3):

1. Descriptive statistics [10, 11] including mean, median, standard deviation (SD) and interquartile range (IQR) and proportions (%) for the ten first quantitative measures ofCD4^+^ T-cell counts and categorized VLs (above or below assay lower limit) after cART;
2. Pearson correlation coefficient [12] and Bland-Altman agreement analysis [13] for quantitative measures of lowest CD4+ T-cell count and log of highest VL before cART;
3. Kappa coefficient agreement [14] for categorized measures of lowest CD4+ T-cell count before cART (< 200 cell/mm^3^, 200 |-- 350 cell/mm^3^, ≥ 350 cell/mm^3^) and highest VL before cART (above or below assay lower limit);

Qualitative or categorized measures are based in quantitative measures considering de following: for the lowest CD4+ T-cell before cART (< 200 cell/mm3, 200 |-- 350 cell/mm3, ≥ 350 cell/mm3) and for the higher VL before cART (above or below assay lower limit).

The database was analyzed with the Statistical Package for the Social Sciences (SPSS) 24 for Windows (International Business Machines Corp, New York, USA) and R version 3.0.3 (http://www.r-project.org/).

## Results

From 8674 enrolled individual in HIV-BCS 8007 were considered eligible for this study. Total follow-up time was 32,397 years. The median duration of follow-up was 3.51 years (IQR 1.63–6.13 years; maximum 11.51 years). As shown in Table 1, 80,302 CD4+ T-cell and 79,997 VL examination records in HIV-BCS versus 94,083 CD4+ T-cell, and 84,810 VLs from SISCEL were observed. The general CD4+ T-cell HIV-BCS testing rate was 247 per 100 py versus 290 per 100 py and the VL HIV-BCS testing rate was 246 per 100 py versus 261 per 100 py. Sites with the lowest CD4+ T-cell HIV-BCS and VL testing rates, respectively, were Manaus – FMT (158 per 100 py and 142 per 100 py) and Belém UREDIP (186 per 100 py and 179 per 100 py). Rio de Janeiro IPEC showed the highest testing rates (186 per 100 py and 179 per 100 py).

**Table 1:** Patients, total of follow-up time (years), number and testing rate of CD4+ T-cell counts and HIV viral load assessments for HIV-BCS and SISCEL databases

Sites of HIV-BCS^a^	N	Follow-up time	Total CD4+ T-cell HIV-BCS	CD4+ T-cell HIV-BCS rate^b^	Total CD4+ T-cell SISCEL	CD4+ T-cell SISCEL rate^b^	Total viral load HIV-BCS	Viral load HIV-BCS rate^b^	Total viral load SISCEL	Viral load SISCEL rate^b^
I	518	1520	2404	158	5869	385	2164	142	3154	207
II	618	2395	4463	186	9424	393	4297	179	6930	289
III	134	342	714	208	1411	412	671	196	789	230
IV	53	57	153	266	287	499	171	297	227	395
V	50	103	255	246	505	487	208	200	309	298
VI	410	1486	3694	248	4263	286	3735	251	4324	290
VII	290	1114	2379	213	4679	419.85	2278	204	3597	322
VIII	1.906	5985	19,274	322	14,251	238.08	19,902	332	12,925	215
IX	519	2320	5389	232	5367	231.24	5288	227	6404	275
X	949	4538	12,005	264	12,691	279.63	12,094	266	15,801	348
XI	847	4881	10,985	225	12,907	264.41	10,940	224	9612	196
XII	408	2177	4974	228	6538	300.23	5022	230	5945	273
XIII	1.305	5472	13,613	248	15,891	290.37	13,227	241	14,793	270
Total	8.007	32,397	80,302	247	94,083	290	79,997	246	84,810	261

^a^I - Manaus – FMT; II - Belém UREDIP; III - Santarém - Municipal STF; IV - Recife - HC/UFPE; V - J. Guararapes - MUNICIPAL STF; VI - Salvador – HUPES; VII - Salvador - CEDAP; VIII - Rio de Janeiro – IPEC; IX Belo Horizonte - UFMG; X - São Paulo - CRT/SP; XI - São Paulo - Municipal Network; XII - SAE S.J.R.P - MUNICIPAL STF; XIII - Porto Alegre – PARTENON

^b^Testing rate per 100 person years

Proportions of CD4^+^ T-cell counts and VL assessments between the two databases are showed in Table 2. In the SISCEL database, missing data were more frequent than in the HIV-BCS for CD4+ T-cell (26.9% [95%CI 25.9 to 27.8%]) versus (7.4% [95%CI 6.9 to 8.0%]) and VL (35.0% [95%CI 33.9 to 36.0%]) versus (12.2% [95%CI 11.5 to 12.9%]) and the proportions look different. However, when missing data were disconsidered, proportions result similar and overlapping intervals occur in all categories. Additional file 1: Tables S1 and S2 show results according to site.

**Table 2:** Proportion and 95% confidence interval for categorized measures of lowest CD4+ T-cell count and highest HIV viral load before cART for HIV-BCS and SISCEL databases

	HIV-BCS		SISCEL	
	N	% (95%CI)	N	% (95%CI)
Full data				
CD4+ T-cell count (cell/mm3)				
< 200	3878	48.4 (47.3–49.6)	2857	35.7 (34.7–36.7)
200 |-- 350	2722	34.0 (32.9–35.0)	2194	27.4 (26.4–28.4)
> 350	812	10.1 (9.5–10.8)	803	10.0 (9.3–10.8)
NDA	595	7.4 (6.9–8.0)	2153	26.9 (25.9–27.8)
Total	8007		8007	
HIV viral load^a^				
Below	188	2.3 (2.0–2.7)	115	1.4 (1.2–1.7)
Above	6844	85.5 (84.8–86.3)	5093	63.6 (62.6–64.6)
NDA	975	12.2 (11.5–12.9)	2799	35.0 (33.9–36.0)
Total	8007		8007	
Disconsidering NDA				
CD4+ T-cell count (cell/mm3)				
< 200	2923	50.8 (49.5–52.1)	2811	48,9 (47.6–50.1)
200 |-- 350	2131	37.0 (35.8–38.2)	2166	37.6 (36.4–38.9)
> 350	699	12.2 (11.3–13.0)	776	13.5 (12.6–14.4)
Total	5753		5753	
HIV viral load^a^				
Below	114	2.2 (1.8–2.6)	115	2.2 (1.8–2.6)
Above	5094	97.8 (97.4–98.2)	5093	97.8 (97.4–98.2)
Total	5208		5208	

95% CI was based on 1000 bootstrap samples

*NDA* No data available

^a^Viral load was grouped in: above or below assay lower limit of detection. The lower limit of detection varied according to method over the years between 400 and 40 copies / ml

High proportion of missing data in the SISCEL database for the lowest CD4+ T-cell count (26.9%) and the highest HIV VL (35%) before cART, were concentrated in the first years of patients inclusion: (1) for the CD4+ T-cell count – 33.9% in 2003 and 82.0% accumulated between 2003 and 2008; and (2) for the highest HIV VL – 32,3% in 2003 and 81.3% accumulated between 2003 and 2008.

The distribution of CD4+ T-cell counts was similar between the two databases: CD4+ T-cell count 1 with mean 334 cells/mm^3^ (SD 204 cells/mm^3^) in HIV-BCS versus 356 cells/mm^3^ (SD 216 cells/mm^3^) in SISCEL database. The proportion of detectable in the first count shows + 1.6% for HIV-BCS (Table 3). In this table we also show increase based in mean ofCD4+ T-cell counts (+ 197 cells/mm^3^ in HIV-BCS versus + 157 cells/mm^3^ in SISCEL) and a decrease in proportion of detectable HIV viral loads (− 17.3% HIV-BCS versus − 12.9% in SISCEL) were shown in data from the two databases. Additional file 1: Table S3 shows the results for each site.

**Table 3:** Descriptive statistics including mean, median, standard deviation (SD) and interquartile range (IQR) for the ten first quantitative measures for CD4^+^ T-cells and proportion of detectable viral load after cART for HIV-BCS and SISCEL databases

	HIV-BCS			SISCEL		
	CD4^+^ T-cell		HIV viral load^a^	CD4^+^ T-cell		HIV viral load^a^
	Mean (SD)	Median (IQR)	Above (%)	Mean (SD)	Median (IQR)	Above (%)
Count 1	334 (204)	307 (189–435)	29.5	356 (216)	327 (198–469)	27.9
Count 2	366 (207)	341 (215–476)	19.5	382 (219)	355 (220–499)	21.5
Count 3	397 (221)	369 (238–515)	18.0	412 (235)	381 (242–538)	17.6
Count 4	425 (235)	395 (262–548)	16.9	433 (241)	403 (259–565)	17.4
Count 5	444 (239)	418 (278–574)	16.1	451 (248)	423 (272–595)	16.9
Count 6	461 (246)	427 (288–597)	15.8	465 (254)	433 (285–607)	16.7
Count 7	480 (252)	455 (303–621)	14.3	478 (260)	445 (293–630)	16.2
Count 8	495 (258)	465 (311–638)	14.1	488 (264)	460 (299–643)	16.1
Count 9	513 (261)	486 (332–657)	12.8	502 (270)	474 (308–659)	15.5
Count 10	531 (272)	503 (336–690)	12.2	515 (282)	482 (314–669)	15.0

^a^Above lower limit of detection. The lower limit of detection varied according to method over the years between 400 and 40 copies / ml

The general correlation observed for the quantitative lowest CD4+ T-cell count before cART was 0.970 (*p* < 0.001); the sites with the lowest and highest correlation were Recife – HC/UFPE - 0.921 (*p* < 0.001) and J. Guararapes – MUNICIPAL STF - 0.997 (*p* < 0.001). For the log of the highest VL before cART, the overall correlation was 0.971 (*p* < 0.001), and Rio de Janeiro – IPEC with 0.950 (*p* < 0.001) and Manaus – FMT 0.997 (*p* < 0.001) accounted for the lowest and highest correlations. In all cases the mean differences between the two data sources were statistically zero (Table 4).

**Table 4:** Pearson coefficient correlation (r) and Bland-Altman agreement analysis for quantitative measures of lowest CD4+ T-cell count and highest viral load before cART between HIV-BCS and SISCEL databases

Sites of HIV-BCS^a^	CD4^+^ T-cell					HIV viral load (log)				
	Correlation		Agreement			Correlation		Agreement		
	r	*p*-value	Mean Diff	CI95%^c^	*p*-value#	r	*p*-value	Mean Diff	CI95%^c^	*p*-value#
I	0.994	< 0.001	0.16	− 1.22 to 1.55	0.815	0.997	< 0.001	0.0110	−0.040 to 0.017	0.429
II	0.974	< 0.001	0.14	−0.67 to 0.95	0.733	0.990	< 0.001	−0.0026	−0.032 to 0.027	0.859
III	0.956	< 0.001	0.71	−2.96 to 4.39	0.701	0.979	< 0.001	0.0075	−0.048 to 0.063	0.790
IV	0.921	< 0.001	0.00	^b^	^b^	0.994	< 0.001	− 0.0040	−0.039 to 0.031	0.822
V	0.997	< 0.001	0.00	^b^	^b^	0.995	< 0.001	− 0.0100	−0.031 to 0.011	0.327
VI	0.990	< 0.001	1.38	−1.7 to 4.51	0.386	0.965	< 0.001	−0.0110	− 0.040 to 0.018	0.453
VII	0.995	< 0.001	−0.03	−0.11 to 0.38	0.318	0.989	< 0.001	−0.0026	−0.036 to 0.035	0.989
VIII	0.956	< 0.001	−0.02	−2.17 to 2.12	0.981	0.950	< 0.001	−0.0050	−0.020 to 0.009	0.466
IX	0.946	< 0.001	0.85	−2.02 to 3.70	0.562	0.973	< 0.001	−0.0170	− 0.051 to 0.016	0.312
X	0.978	< 0.001	0.84	−1.85 to 3.54	0.540	0.965	< 0.001	0.0060	−0.010 to 0.030	0.634
XI	0.942	< 0.001	−0.96	−3.51 to 1.59	0.460	0.975	< 0.001	0.0030	−0.014 to 0.021	0.686
XII	0.967	< 0.001	−0.50	−1.5 to 0.58	0.361	0.990	< 0.001	0.0040	−0.027 to 0.035	0.800
XIII	0.971	< 0.001	−0.36	−2.00 to 1.33	0.673	0.966	< 0.001	0.0110	−0.017 to 0.020	0.903
Total	0.970	< 0.001	0.073	−0.07 to 0.88	0.861	0.971	< 0.001	−0.0025	−0.010 to 0.005	0.509

# Null hypothesis means (DIFF) = 0

^a^I - Manaus – FMT; II - Belém UREDIP; III - Santarém - Municipal STF; IV - Recife - HC/UFPE; V - J. Guararapes - MUNICIPAL STF; VI - Salvador – HUPES; VII - Salvador - CEDAP; VIII - Rio de Janeiro – IPEC; IX Belo Horizonte - UFMG; X - São Paulo - CRT/SP; XI - São Paulo - Municipal Network; XII - SAE S.J.R.P - MUNICIPAL STF; XIII - Porto Alegre – PARTENON;

^b^Statistics were not calculated because the standard deviation is zero;

^c^Including upper and higher limits of the confidence interval CI95%

The overall agreement coefficients for categorized CD4+ T-cell counts - 0.932 (*p* < 0.001) and HIV VLs - 0.996 (*p* < 0.001) were high. The Municipal Network in São Paulo presented the lowest kappa coefficient for both biomarkers - 0.867 (*p* < 0.001) for CD4^+^ T-cell counts and 0.855 (*p* < 0.001) for HIV VLs. The highest kappa agreement (1.00) was observed in J. Guararapes – MUNICIPAL STF for both indicators (Table 5).

**Table 5:** Kappa coefficient agreement for categorized measures of lowest CD4+ T-cell count before cART and highest viral load before cART between HIV-BCS and SISCEL databases

Sites of HIV-BCS^a^	CD4^+^ T-cell^b^		Viral load^c^	
	Kappa	*p*-value	Kappa	*p*-value
I	0.980	*P* < 0.001	1.000	*P* < 0.001
II	0.983	*P* < 0.001	1.000	*P* < 0.001
III	0.905	*P* < 0.001	1.000	*P* < 0.001
IV	0.889	*P* < 0.001	1.000	*P* < 0.001
V	1.000	*P* < 0.001	1.000	*P* < 0.001
VI	1.000	*P* < 0.001	1.000	*P* < 0.001
VII	0.985	*P* < 0.001	1.000	*P* < 0.001
VIII	0.885	*P* < 0.001	1.000	*P* < 0.001
IX	0.938	*P* < 0.001	1.000	*P* < 0.001
X	0.937	*P* < 0.001	1.000	*P* < 0.001
XI	0.867	*P* < 0.001	0.855	*P* < 0.001
XII	0.989	*P* < 0.001	1.000	*P* < 0.001
XIII	0.940	*P* < 0.001	1.000	*P* < 0.001
Total	0.932	*P* < 0.001	0.996	*P* < 0.001

^a^I - Manaus – FMT; II - Belém UREDIP; III - Santarém - Municipal STF; IV - Recife - HC/UFPE; V - J. Guararapes - MUNICIPAL STF; VI - Salvador – HUPES; VII - Salvador - CEDAP; VIII - Rio de Janeiro – IPEC; IX Belo Horizonte - UFMG; X - São Paulo - CRT/SP; XI - São Paulo - Municipal Network; XII - SAE S.J.R.P - MUNICIPAL STF; XIII - Porto Alegre – PARTENON;

^b^Categorized lowest CD4+ T-cell before cART was grouped in: < 200 cell/mm3, 200 |-- 350 cell/mm3, ≥ 350 cell/mm3;

^c^Viral load was grouped in: above or below assay lower limit of detection. The lower limit of detection varied according to method over the years between 400 and 40 copies / ml

## Discussion

CD4^+^ T-cell and VL counts are two biomarkers of responses to antiretroviral treatment and HIV disease progression that have been used to monitor HIV infection in clinical follow-up. The VL is the most important indicator of initial and sustained response to ART and should be measured in all patients infected with HIV on entry into treatment, at the onset of therapy and on a regular basis thereafter. The CD4 count is the most important laboratory indicator of immune status in HIV-infected patients. It is also the strongest predictor of HIV disease progression and subsequent survival rate, according to results of clinical trials and cohort studies [15–18].

This study proposed a validation method based on the correlation and agreement coefficient for VL and CD4^+^ T-cell data from the HIV-BCS and SISCEL databases. Our interest was not to evaluate viral suppression or the immunerestoration provided by antiviral therapy, since this had been widely described in the literature [19–21]. The main focus of this paper was to show that information from HIV/AIDS medical records has proven to be of high quality when compared with the same data obtained from other systems. The main difference between these two sources of information (HIV-BCS versus SISCEL databases) is based on an increased likelihood of interference of human intervention in the first database, especially in phase 1, that used paper questionnaires to collect information from the patient’s medical record (Fig. 1).

This paper also allowed us to study the distribution of CD4^+^ T-cell counts and HIV VL assessments according to research sites, and in this regard we obtained important findings. About the results presented in Table 1, sites such as Manaus – FMT and Belém – UREDIP, located in the north of Brazil had a low density of laboratory tests in the HIV-BCS database (less than 2 measurements per persons annually for CD4^+^ T-cell and VL), but when these sites were observed in the SISCEL database, they presented frequencies of testing that were comparable to that seen in the other sites (more than 3 per year). This can be explained by the incompleteness of medical records as far as laboratory results are concerned. In contrast, Rio de Janeiro – IPEC showed more CD4^+^ T-cell and VL records in the HIV/BSC database (more than 3 per year for CD4^+^ T-cell and VL) than in SISCEL, as this site is a major reference center for in HIV research and clinical care. Furthermore, their laboratory integrates the National Network of Laboratories for CD4^+^ T-cell, VL and HIV genotyping tests and as they have a large and busy outpatient clinic, we suggest an internal flow that provides CD4^+^ T-cell and VL results for clinicians could have led to the partial feeding of the SISCEL database.

Vieira and Garrett [14] suggested operational cutoff points for kappa coefficient: less than chance agreement (< 0); slight agreement (0.01 to 0.20); fair agreement (0.21 to 0.40); moderate agreement (0.41 to 0.60); substantial agreement (0.61 to 0.80); and almost perfect agreement (0.81to 1.00). Mukaka [12] also suggested the rule of thumb for interpreting the size of a correlation coefficient: negligible correlation (0.0 to 0.30); low correlation (0.30 to 0.50); moderate correlation (0.50 to 0.80); high correlation (0.70 to 0.90); and very high correlation (0.90 to 1.00). Based on these authors, we feel comfortable saying that this study showed there were high correlation and agreement for CD4^+^ T-cell counts and VL assessments, taking overall and per site data.

In previous reports authors [5–7] have compared data from HIV surveillance information systems with those obtained from medical records or independent databases. They found substantial or almost perfect agreement for age, race, and gender, but poorer agreement for mode of HIV acquisition, CD4 + cell counts, and the more complex categorization of AIDS case definition.

Moreover, studies have been conducted to validate self-reported health information versus registered information from medical records. For instance, Kalichman, Rompa and Cage [22] found good agreement in self-reporting of CD4 cell counts, but not for HIV VLs. In a marginalized population in particular, agreement between self-reports and medical records was poor for ambulatory visits, poor to fair for medication use, and poor for laboratory tests. However, the agreement for CD4 count was substantially better [23]. Using another strategy An et al. [24] showed an agreement between self-reported and medical records was good in HIV status and date of first positive HIV test, but poor in date of last negative HIV test.

Two implications can be realized from this study: (1) concerning the HIV/AIDS cohort study in Brazil, we believe no additional transcription of CD4+ T-cell and VL counts from patients’ medical records is necessary, due to a reliable quality in the SISCEL database; (2) and another concerning the health service, with high correlation and agreement of their data, the permanent evaluation of the therapeutic success of the patients can be accomplished, and it is not necessary to carry out specific studies for this purpose.

The main strength of our study relies on the linkage of strongly robust HVI/BCS databases with 8007 subjects and with a long follow-up time (32,397.12 years) with thousands of tests results accumulated over the year sand the SISCEL database that integrates the National HIV/AIDS register in Brazil. All T-cell counts (CD4^+^/CD8^+^) and HIV VL assessments performed in the National Network of Laboratories (NNT) are mandatorily registered in this database.

Despite these results, some limitations of our study should be pointed out. We realize that high proportion of missing data in the SISCEL database for thelowest CD4+ T-cell count and the highest HIV VL before cART, were concentrated in the first years of patients inclusion. The SISCEL system has been effectively in since 2002 and, although a study has already shown its good quality for epidemiological surveillance [20], so it is fair to speculate that it did not perform very well in the first years of use.

We encourage further studies with SISCEL system in order to verify the quality information improvement in the time and new studies including another national database like SICLOM – Logistic Control System Drugs, SINAN – Notifiable Diseases Information System (surveillance system) or SIM – Mortality Information System and HIV-BCS. Validation data of socio-demographic characteristics, loss to follow-up, mortality and cART schemes can be evaluated.

## Conclusion

The current study confirms that CD4^+^ T-cell counts and HIV VL assessments from HIV-BCS have high correlation and agreement with data obtained from SISCEL, especially after exclusion of missing data. The HIV-BCS database has a lower proportion of missing data concerning CD4^+^ T-cell counts and HIV VL, as compared to SISCEL.

## Additional file

Additional file 1: **Table S1.** Proportion and 95% confidence interval for qualitative measures of lowest CD4+ T-cell count before cART per site. **Table S2** Proportion and 95% confidence intervals for qualitative measures of viral load before cART per site. **Table S3.** Descriptive statistics including mean, median, standard deviation (SD) and interquartile range (IQR) for the ten first quantitative measures for CD4^+^ T-cell and viral load after cART. (DOCX 101 kb)

## Abbreviations

cART: Combination Antiretroviral Therapy
CD4+ T-cell: CD4+ T-cell lynphocytes
HIV: Human immunodeficiency virus
HIV-BCS: HIV-Brazil cohort study
IPEC: Instituto de Pesquisas Clínicas Evandro Chagas (Evandro Chagas Institute of Clinical Research)
IQR: Interquartile range
IRB: Institutional Review Boards
NNT: National Network of Laboratories
Py: Person-year
SICLOM: Sistema de Controle Logístico de Medicamentos (Medication Logistics Control System)
SIM: Sistema de Informação sobre Mortalidade (Mortality Information System)
SINAN: Sistema de Informação de Agravos de Notificação (Notifiable Diseases Information System)
SISCEL: Brazilian Laboratory Tests Control System
