# Supplementary material for: Validation of CD4+ T-cell and viral load data from the HIV-Brazil Cohort Study using secondary system data
Source: BMC Infect Dis. 2018 Dec 4;18:617. doi: 10.1186/s12879-018-3536-4 (PMC6278123; doi:10.1186/s12879-018-3536-4)
Supplement: Supplementary file 1 — Table S1. Proportion and 95% confidence interval for qualitative measures of lowest CD4+ T-cell count before cART per site. Table S2 Proportion and 95% confidence intervals for qualitative measures of viral load before cART per site. Table S3. Descriptive statistics including mean, median, standard deviation (SD) and interquartile range (IQR) for the ten first quantitative measures for CD4+ T-cell and viral load after cART. (DOCX 101 kb) [file 12879_2018_3536_MOESM1_ESM.docx]

**ADDITIONAL FILE 1**

VALIDATION OF CD4+ T-CELL AND VIRAL LOAD DATA FROM THE HIV-BRAZIL COHORT STUDY USING SECONDARY SYSTEM DATA

**Table S1** Proportion and 95% confidence interval for qualitative measures of lowest CD4+ T-cell count before cART per site

|  | CD4+ T-cell (mm^3^) | **HIV/CBS** | | **SISCEL** | |
| --- | --- | --- | --- | --- | --- |
|  |  | **N** | **% (95%CI)** | **N** | **% (95%CI)** |
| I - Manaus - FMT | <200 | 318 | 61.3 (57.3-65.8) | 299 | 57.7 (53.4-61.9) |
|  | 200 \|-- 350 | 75 | 14.4 (11.5-17.5) | 75 | 14.4 (11.5-17.5) |
|  | >350 | 20 | 3.86 (2.31-5.59) | 23 | 4.4 (2.9-6.4) |
|  | NDA | 105 | 20.2 (16.9-23.7) | 121 | 23.3 (19.8-26.8) |
|  | Total | 518 | 100 (100-100) | 518 | 100 (100-100) |
| II - Belém UREDIP | <200 | 400 | 64.7 (60.5-68.2) | 393 | 63.5 (59.7-67.3) |
|  | 200 \|-- 350 | 153 | 24.7 (21.3-28.1) | 149 | 24.1 (20.5-27.6) |
|  | >350 | 23 | 3.72 (2.26-5.33) | 20 | 3.2 (1.9-4.9) |
|  | NDA | 42 | 6.79 (5.01-8.89) | 56 | 9.1 (7-11.4) |
|  | Total | 618 | 100 (100-100) | 618 | 100 (100-100) |
| III - Santarém - Municipal STF | <200 | 75 | 55.9 (46.2-64.1) | 60 | 44.7 (35.8-53.7) |
|  | 200 \|-- 350 | 28 | 20.8 (14.1-28.3) | 24 | 17.9 (11.9-24.6) |
|  | >350 | 8 | 5.97 (2.23-10.4) | 6 | 4.5 (1.5-8.2) |
|  | NDA | 23 | 17.1 (11.2-23.8) | 44 | 32.8 (24.6-41) |
|  | Total | 134 | 100 (100-100) | 134 | 100 (100-100) |
| IV - Recife - HC/UFPE | <200 | 19 | 35.8 (22.6-49) | 21 | 39.6 (26.4-52.8) |
|  | 200 \|-- 350 | 21 | 39.6 (26.4-52.8) | 18 | 33.9 (22.6-47.1) |
|  | >350 | 8 | 15 (5.66-26.3) | 8 | 15 (5.7-24.5) |
|  | NDA | 5 | 9.43 (1.88-16.9) | 6 | 11.3 (3.8-20.7) |
|  | Total | 53 | 100 (100-100) | 53 | 100 (100-100) |
| V – Jab. dos Guararapes - MUNICIPAL STF | <200 | 28 | 56 (42-70) | 27 | 54 (40-68) |
|  | 200 \|-- 350 | 17 | 34 (22-46) | 17 | 34 (22-48) |
|  | >350 | 4 | 8 (2-16) | 3 | 6 (0-14) |
|  | NDA | 1 | 2 (0-6) | 3 | 6 (0-14) |
|  | Total | 50 | 100 (100-100) | 50 | 100 (100-100) |
| VI - Salvador - HUPES | <200 | 211 | 51.4 (46-56) | 182 | 44.3 (39.7-49) |
|  | 200 \|-- 350 | 109 | 26.5 (22.6-31.2) | 94 | 22.9 (19-27) |
|  | >350 | 72 | 17.5 (13.9-20.9) | 67 | 16.3 (12.9-19.7) |
|  | NDA | 18 | 4.39 (2.43-6.57) | 67 | 16.3 (12.9-20) |
|  | Total | 410 | 100 (100-100) | 410 | 100 (100-100) |
| VII - Salvador - CEDAP | <200 | 143 | 49.3 (43.4-54.8) | 140 | 48.2 (42-53.7) |
|  | 200 \|-- 350 | 91 | 31.3 (25.8-37.2) | 92 | 31.7 (26.5-37.5) |
|  | >350 | 29 | 10 (6.89-13.4) | 27 | 9.3 (5.9-13.1) |
|  | NDA | 27 | 9.31 (5.86-12.7) | 31 | 10.6 (7.6-14.4) |
|  | Total | 290 | 100 (100-100) | 290 | 100 (100-100) |
| VIII - Rio de Janeiro - IPEC | <200 | 843 | 44.2 (41.8-46.5) | 635 | 33.3 (31-35.5) |
|  | 200 \|-- 350 | 638 | 33.4 (31.3-35.6) | 589 | 30.9 (28.8-33) |
|  | >350 | 235 | 12.3 (10.8-13.9) | 276 | 14.4 (12.8-16.1) |
|  | NDA | 190 | 9.96 (8.65-11.3) | 406 | 21.3 (19.4-23.2) |
|  | Total | 1906 | 100 (100-100) | 1906 | 100 (100-100) |
| IX Belo Horizonte - UFMG | <200 | 233 | 44.8 (40.2-48.9) | 182 | 35 (30.8-39.1) |
|  | 200 \|-- 350 | 217 | 41.8 (37.3-46.4) | 195 | 37.5 (33.5-42) |
|  | >350 | 43 | 8.28 (5.97-10.7) | 44 | 8.5 (5.8-11.1) |
|  | NDA | 26 | 5 (3.27-6.93) | 98 | 18.8 (15.6-21.9) |
|  | Total | 519 | 100 (100-100) | 519 | 100 (100-100) |
| X - São Paulo - CRT/SP | <200 | 405 | 42.6 (39.6-46) | 324 | 34.1 (31-37.1) |
|  | 200 \|-- 350 | 368 | 38.7 (35.7-41.8) | 321 | 33.8 (30.8-36.9) |
|  | >350 | 157 | 16.5 (14.2-18.8) | 143 | 15 (12.9-17.4) |
|  | NDA | 19 | 2 (1.15-3.05) | 161 | 16.9 (14.6-19.2) |
|  | Total | 949 | 100 (100-100) | 949 | 100 (100-100) |
| XI - SAE S.J.R. Preto - MUNICIPAL STF | <200 | 228 | 55.8 (51.2-61) | 191 | 46.8 (42.1-51.7) |
|  | 200 \|-- 350 | 139 | 34 (29.4-38.7) | 121 | 29.6 (25.2-34) |
|  | >350 | 23 | 5.63 (3.43-7.59) | 22 | 5.4 (3.2-7.4) |
|  | NDA | 18 | 4.41 (2.45-6.61) | 74 | 18.1 (14.4-22.2) |
|  | Total | 408 | 100 (100-100) | 408 | 100 (100-100) |
| XII - São Paulo - Municipal Network | <200 | 429 | 50.6 (47.3-54) | 144 | 17 (14.5-19.9) |
|  | 200 \|-- 350 | 229 | 27 (24-30.1) | 98 | 11.5 (9.6-13.6) |
|  | >350 | 86 | 10.1 (8.14-12.5) | 58 | 6.8 (5.2-8.9) |
|  | NDA | 103 | 12.1 (9.91-14.4) | 547 | 64.5 (61.1-67.7) |
|  | Total | 847 | 100 (100-100) | 847 | 100 (100-100) |
| XIII - Porto Alegre - PARTENON | <200 | 546 | 41.8 (39.1-44.5) | 259 | 19.8 (17.6-21.9) |
|  | 200 \|-- 350 | 637 | 48.8 (46.1-51.7) | 401 | 30.7 (28.3-33.1) |
|  | >350 | 104 | 7.96 (6.59-9.5) | 106 | 8.1 (6.7-9.6) |
|  | NDA | 18 | 1.37 (0.76-2.06) | 539 | 41.3 (38.6-44.1) |
|  | Total | 1305 | 100 (100-100) | 1305 | 100 (100-100) |

NDA - No data avaliable

HIV/BVS - HIV Cohort Brazilian Study

SISCEL - Laboratory Tests Control System

95% CI was based on 1.000 bootstrap samples

**Table** **S2** Proportion and 95% confidence intervals for qualitative measures of viral load before cART per site

|  |  | **HIV/CBS** | | **SISCEL** | |
| --- | --- | --- | --- | --- | --- |
|  |  | **N** | **% (95%CI)** | **N** | **% (95%CI)** |
| I - Manaus - FMT | Below | 29 | 5.59 (3.66-7.52) | 28 | 5.4 (3.47-7.33) |
|  | Above | 360 | 69.4 (65.4-73.3) | 327 | 63.1 (59-67.3) |
|  | NDA | 129 | 24.9 (21-28.5) | 163 | 31.4 (27.4-35.3) |
|  | Below | 518 | 100 (100-100) | 518 | 100 (100-100) |
| II - Belém UREDIP | Below | 6 | 0.97 (0.32-1.94) | 5 | 0.8 (0.16-1.61) |
|  | Above | 518 | 83.8 (80.9-86.5) | 490 | 79.2 (75.8-82.3) |
|  | NDA | 94 | 15.2 (12.4-18.1) | 123 | 19.9 (16.9-23.4) |
|  | Below | 618 | 100 (100-100) | 618 | 100 (100-100) |
| III - Santarém - Municipal STF | Below | 5 | 3.73 (0.74-6.71) | 2 | 1.49 (0-3.73) |
|  | Above | 92 | 68.6 (60.4-76.1) | 71 | 52.9 (44.7-61.9) |
|  | NDA | 37 | 27.6 (20.1-35) | 61 | 45.5 (36.5-53.7) |
|  | Below | 134 | 100 (100-100) | 134 | 100 (100-100) |
| IV - Recife - HC/UFPE | Below | 5 | 9.43 (1.88-16.9) | 5 | 9.43 (1.88-16.9) |
|  | Above | 41 | 77.3 (66-88.6) | 36 | 67.9 (54.7-79.2) |
|  | NDA | 7 | 13.2 (5.66-22.6) | 12 | 22.6 (11.3-33.9) |
|  | Below | 53 | 100 (100-100) | 53 | 100 (100-100) |
| V – Jab. dos Guararapes - MUNICIPAL STF | Below | 3 | 6 (0-14) | 3 | 6 (0-14) |
|  | Above | 39 | 78 (66-88) | 36 | 72 (58-84) |
|  | NDA | 8 | 16 (8-27.9) | 11 | 22 (12-34) |
|  | Below | 50 | 100 (100-100) | 50 | 100 (100-100) |
| VI - Salvador - HUPES | Below | 6 | 1.46 (0.48-2.68) | 2 | 0.48 (0-1.21) |
|  | Above | 375 | 91.4 (88.5-94.1) | 330 | 80.4 (76.8-84.1) |
|  | NDA | 29 | 7.07 (4.63-9.51) | 78 | 19 (15.3-22.6) |
|  | Below | 410 | 100 (100-100) | 410 | 100 (100-100) |
| VII - Salvador - CEDAP | Below | 9 | 3.1 (1.37-5.17) | 6 | 2.06 (0.68-3.79) |
|  | Above | 244 | 84.1 (79.6-88.2) | 221 | 76.2 (71.7-81) |
|  | NDA | 37 | 12.7 (9.31-16.5) | 63 | 21.7 (17.2-26.5) |
|  | Below | 290 | 100 (100-100) | 290 | 100 (100-100) |
| VIII - Rio de Janeiro - IPEC | Below | 36 | 1.88 (1.31-2.51) | 31 | 1.62 (1.1-2.2) |
|  | Above | 1573 | 82.5 (80.8-84.2) | 1255 | 65.8 (63.6-67.9) |
|  | NDA | 297 | 15.5 (13.9-17.3) | 620 | 32.5 (30.3-34.8) |
|  | Below | 1906 | 100 (100-100) | 1906 | 100 (100-100) |
| IX Belo Horizonte - UFMG | Below | 12 | 2.31 (1.15-3.66) | 8 | 1.54 (0.57-2.69) |
|  | Above | 430 | 82.8 (79.5-85.7) | 371 | 71.4 (67.6-75.3) |
|  | NDA | 77 | 14.8 (11.9-17.9) | 140 | 26.9 (23.1-30.4) |
|  | Below | 519 | 100 (100-100) | 519 | 100 (100-100) |
| X - São Paulo - CRT?/SP | Below | 11 | 1.15 (0.52-1.79) | 7 | 0.73 (0.21-1.26) |
|  | Above | 900 | 94.8 (93.3-96.2) | 724 | 76.2 (73.3-78.7) |
|  | NDA | 38 | 4 (2.84-5.47) | 218 | 22.9 (20.5-25.8) |
|  | Below | 949 | 100 (100-100) | 949 | 100 (100-100) |
| XI - SAE S.J.R. Preto - MUNICIPAL STF | Below | 18 | 4.41 (2.45-6.37) | 7 | 1.71 (0.73-2.94) |
|  | Above | 365 | 89.4 (86.5-92.4) | 297 | 72.7 (68.3-76.7) |
|  | NDA | 25 | 6.12 (3.92-8.57) | 104 | 25.4 (21.5-30.1) |
|  | Below | 408 | 100 (100-100) | 408 | 100 (100-100) |
| XII - São Paulo - Municipal Network | Below | 30 | 3.54 (2.36-4.72) | 4 | 0.47 (0.11-0.94) |
|  | Above | 689 | 81.3 (78.7-83.9) | 188 | 22.1 (19.4-25.2) |
|  | NDA | 128 | 15.1 (12.6-17.5) | 655 | 77.3 (74.2-79.9) |
|  | Below | 847 | 100 (100-100) | 847 | 100 (100-100) |
| XIII - Porto Alegre - PARTENON | Below | 18 | 1.37 (0.76-2.06) | 7 | 0.53 (0.22-0.91) |
|  | Above | 1218 | 93.3 (91.8-94.6) | 747 | 57.2 (54.4-59.9) |
|  | NDA | 69 | 5.28 (4.06-6.59) | 551 | 42.2 (39.5-45) |
|  | Total | 1305 | 100 (100-100) | 1305 | 100 (100-100) |

NDA – No data available

HIV/BVS - HIV Cohort Brazilian Study

SISCEL - Laboratory Tests Control System

95% CI was based on 1.000 bootstrap samples

¥ Viral load was grouped in: above or below assay lower limit of detection. The lower limit of detection varied according to method over the years between 400 and 40 copies / ml.

**Table** **S3** Descriptive statistics including mean, median, standard deviation (SD) and interquartile range (IQR) for the ten first quantitative measures for CD4^+^ T-cell and viral load after cART

|  | Count | **HIV/CBS** | | | | | | **SISCEL** | | | | | |
| --- | --- | --- | --- | --- | --- | --- | --- | --- | --- | --- | --- | --- | --- |
| Site |  | CD4^+^ T-cell | | | | | **VL** | CD4^+^ T-cell | | | | | **VL** |
|  |  | **Med** | **SD** | **M** | **P25** | **P75** | **Det (%)** | **Med** | **SD** | **M** | **SD** | **Mean** | **Det (%)** |
| I | 1 | 259.0 | 173.8 | 230.0 | 144.5 | 330.3 | 32.3 | 255.3 | 156.8 | 230.0 | 142.0 | 329.8 | 29.3 |
|  | 2 | 289.3 | 201.0 | 264.0 | 157.3 | 379.0 | 20.3 | 278.3 | 174.5 | 250.0 | 150.3 | 371.8 | 25.3 |
|  | 3 | 326.9 | 231.1 | 289.5 | 174.8 | 420.5 | 16.0 | 302.8 | 193.4 | 267.0 | 158.0 | 417.0 | 17.8 |
|  | 4 | 339.7 | 215.1 | 313.0 | 189.0 | 438.0 | 12.0 | 318.5 | 197.1 | 296.0 | 171.0 | 430.0 | 16.0 |
|  | 5 | 348.7 | 225.4 | 319.0 | 195.0 | 437.0 | 9.5 | 330.7 | 214.4 | 297.5 | 175.0 | 449.0 | 13.3 |
|  | 6 | 360.8 | 244.0 | 320.0 | 200.0 | 457.5 | 8.7 | 336.8 | 214.0 | 301.0 | 175.0 | 458.0 | 11.8 |
|  | 7 | 355.7 | 213.1 | 319.0 | 188.0 | 480.0 | 4.2 | 361.0 | 228.1 | 320.5 | 199.5 | 470.3 | 9.3 |
|  | 8 | 363.9 | 207.4 | 335.0 | 204.3 | 446.5 | 2.9 | 373.8 | 237.5 | 329.0 | 193.8 | 504.5 | 8.9 |
|  | 9 | 409.8 | 237.1 | 343.0 | 227.0 | 542.0 | 2.1 | 393.5 | 258.1 | 345.0 | 214.0 | 560.0 | 7.1 |
|  | 10 | 374.2 | 194.8 | 346.5 | 227.0 | 525.0 | 1.2 | 397.6 | 246.9 | 356.0 | 217.0 | 529.5 | 6.0 |
| II | 1 | 279.3 | 163.5 | 257.0 | 160.0 | 364.0 | 38.5 | 280.7 | 166.8 | 258.0 | 160.8 | 361.5 | 38.5 |
|  | 2 | 321.3 | 172.7 | 302.0 | 193.0 | 416.0 | 32.0 | 311.7 | 175.2 | 292.0 | 188.0 | 403.0 | 36.9 |
|  | 3 | 353.5 | 199.5 | 327.0 | 213.0 | 454.5 | 28.5 | 339.5 | 182.2 | 317.0 | 209.0 | 442.0 | 28.0 |
|  | 4 | 397.2 | 228.5 | 363.0 | 242.8 | 515.5 | 23.3 | 355.7 | 196.8 | 337.0 | 209.0 | 469.0 | 25.2 |
|  | 5 | 416.1 | 234.3 | 376.0 | 248.0 | 540.0 | 18.1 | 378.3 | 217.0 | 342.5 | 229.5 | 491.8 | 24.4 |
|  | 6 | 421.4 | 247.5 | 394.0 | 255.0 | 553.5 | 15.4 | 397.8 | 231.0 | 368.0 | 232.5 | 517.0 | 23.1 |
|  | 7 | 454.0 | 258.3 | 443.0 | 280.0 | 571.0 | 12.1 | 408.2 | 234.6 | 373.5 | 239.3 | 539.8 | 19.3 |
|  | 8 | 477.9 | 285.0 | 409.0 | 290.0 | 598.0 | 9.1 | 421.9 | 250.5 | 394.0 | 238.0 | 552.0 | 18.3 |
|  | 9 | 493.1 | 263.2 | 462.0 | 316.5 | 645.8 | 4.9 | 431.4 | 260.0 | 396.5 | 249.0 | 566.3 | 14.1 |
|  | 10 | 530.3 | 303.9 | 483.0 | 310.0 | 741.0 | 3.9 | 447.2 | 279.7 | 396.0 | 267.0 | 596.0 | 12.9 |
| III | 1 | 292.4 | 177.3 | 261.5 | 175.8 | 367.0 | 34.3 | 311.5 | 203.9 | 262.0 | 171.5 | 384.5 | 30.6 |
|  | 2 | 316.3 | 166.5 | 280.0 | 193.3 | 412.0 | 23.9 | 323.4 | 179.8 | 281.5 | 182.8 | 457.3 | 21.6 |
|  | 3 | 348.8 | 232.3 | 314.0 | 205.5 | 428.5 | 17.2 | 349.3 | 207.5 | 299.5 | 212.0 | 461.0 | 15.7 |
|  | 4 | 399.8 | 257.0 | 368.0 | 236.3 | 531.3 | 11.2 | 374.1 | 247.8 | 335.0 | 213.0 | 451.5 | 11.9 |
|  | 5 | 376.2 | 188.4 | 368.0 | 242.5 | 506.8 | 6.7 | 379.8 | 196.5 | 363.0 | 212.5 | 501.5 | 8.2 |
|  | 6 | 438.8 | 229.4 | 417.0 | 304.8 | 554.5 | 6.7 | 403.3 | 222.4 | 377.5 | 247.3 | 542.5 | 8.2 |
|  | 7 | 491.2 | 246.9 | 477.0 | 320.0 | 599.0 | 6.0 | 442.7 | 242.7 | 399.0 | 288.0 | 561.0 | 6.0 |
|  | 8 | 446.9 | 216.3 | 399.0 | 268.5 | 620.0 | 3.0 | 432.8 | 210.2 | 412.0 | 289.5 | 561.0 | 3.7 |
|  | 9 | 446.4 | 211.1 | 388.0 | 293.5 | 591.3 | 1.5 | 451.9 | 222.8 | 396.0 | 300.0 | 587.0 | 3.0 |
|  | 10 | 458.1 | 173.3 | 446.0 | 355.0 | 587.0 | 0.7 | 459.9 | 243.6 | 435.0 | 301.5 | 592.5 | 3.0 |
| IV | 1 | 388.1 | 222.7 | 356.0 | 250.0 | 505.0 | 30.2 | 369.5 | 222.0 | 351.0 | 217.3 | 478.5 | 35.8 |
|  | 2 | 453.6 | 254.1 | 394.0 | 328.5 | 541.0 | 5.7 | 385.5 | 252.6 | 346.5 | 230.5 | 476.3 | 20.8 |
|  | 3 | 462.4 | 182.2 | 417.0 | 347.0 | 576.0 | 7.5 | 449.9 | 246.2 | 394.5 | 317.3 | 516.3 | 7.5 |
|  | 4 | 585.2 | 256.5 | 588.0 | 422.5 | 670.5 | 5.7 | 475.4 | 246.2 | 398.5 | 349.8 | 590.3 | 5.7 |
|  | 5 | 589.0 | 304.1 | 522.0 | 412.0 | 993.0 | 1.9 | 474.7 | 235.7 | 408.0 | 361.0 | 623.5 | - |
|  | 6 | 588.0 | 242.5 | 570.0 | 355.0 | - | - | 577.2 | 261.9 | 554.0 | 389.0 | 644.0 | - |
|  | 7 | 505.5 | 142.1 | 505.5 | 405.0 | - | - | 607.4 | 258.4 | 594.0 | 443.0 | 698.8 | - |
|  | 8 | 504.0 | - | 504.0 | 504.0 | 504.0 | - | 620.6 | 207.2 | 593.0 | 473.0 | 808.0 | - |
|  | 9 | - | - | - | - | - | - | 671.1 | 238.6 | 602.0 | 466.0 | 873.0 | - |
|  | 10 | - | - | - | - | - | - | 593.1 | 178.2 | 565.0 | 438.0 | 691.0 | - |
| V | 1 | 319.6 | 190.3 | 273.0 | 197.5 | 424.0 | 38.0 | 311.9 | 187.3 | 271.0 | 194.0 | 423.0 | 38.0 |
|  | 2 | 341.1 | 188.5 | 292.5 | 203.5 | 497.0 | 34.0 | 337.0 | 196.6 | 282.0 | 205.0 | 475.0 | 34.0 |
|  | 3 | 368.4 | 183.1 | 311.0 | 240.0 | 512.0 | 18.0 | 356.1 | 189.3 | 312.5 | 213.0 | 499.3 | 30.0 |
|  | 4 | 392.6 | 172.6 | 360.0 | 272.0 | 547.0 | 18.0 | 373.4 | 192.8 | 335.0 | 249.5 | 511.0 | 22.0 |
|  | 5 | 381.3 | 195.2 | 362.5 | 255.8 | 488.8 | 14.0 | 415.3 | 209.7 | 396.5 | 246.3 | 601.5 | 10.0 |
|  | 6 | 412.8 | 169.7 | 412.0 | 289.5 | 527.3 | 8.0 | 427.7 | 235.5 | 402.5 | 250.0 | 594.5 | - |
|  | 7 | 456.9 | 284.0 | 406.0 | 221.0 | 593.0 | - | 379.7 | 229.8 | 357.0 | 236.0 | 482.0 | - |
|  | 8 | 462.9 | 245.3 | 364.0 | 244.5 | 707.0 | - | 434.0 | 265.2 | 349.0 | 251.5 | 596.5 | - |
|  | 9 | 453.1 | 219.3 | 355.0 | 294.0 | 655.0 | - | 421.0 | 191.5 | 383.0 | 295.0 | 546.5 | - |
|  | 10 | 539.8 | 161.3 | 593.0 | 378.5 | 674.5 | - | 452.8 | 195.1 | 385.0 | 317.8 | 577.0 | - |
| VI | 1 | 376.8 | 273.4 | 327.0 | 188.5 | 501.3 | 29.8 | 397.1 | 286.6 | 335.0 | 187.0 | 534.0 | 29.8 |
|  | 2 | 391.7 | 226.9 | 359.0 | 227.3 | 493.5 | 18.5 | 413.1 | 264.0 | 372.0 | 212.5 | 560.3 | 21.0 |
|  | 3 | 426.7 | 248.5 | 385.0 | 253.0 | 546.0 | 16.8 | 442.3 | 267.1 | 391.0 | 245.0 | 604.0 | 15.6 |
|  | 4 | 448.8 | 254.9 | 417.0 | 282.0 | 589.0 | 14.8 | 481.1 | 289.7 | 432.0 | 257.0 | 666.0 | 13.7 |
|  | 5 | 466.1 | 238.8 | 437.0 | 295.0 | 606.0 | 12.4 | 487.7 | 275.8 | 443.0 | 277.0 | 641.5 | 13.4 |
|  | 6 | 466.1 | 243.9 | 434.0 | 294.0 | 610.0 | 11.2 | 499.4 | 279.9 | 429.5 | 297.3 | 668.8 | 11.7 |
|  | 7 | 477.7 | 252.3 | 467.0 | 327.5 | 600.5 | 10.2 | 504.7 | 259.0 | 458.0 | 340.0 | 647.5 | 12.2 |
|  | 8 | 507.4 | 288.9 | 465.0 | 332.5 | 654.8 | 7.3 | 486.4 | 248.5 | 469.0 | 309.5 | 619.8 | 11.5 |
|  | 9 | 523.9 | 292.6 | 502.0 | 362.5 | 655.0 | 7.6 | 508.5 | 265.7 | 483.5 | 329.0 | 649.3 | 10.5 |
|  | 10 | 540.2 | 269.5 | 538.5 | 375.0 | 701.5 | 8.5 | 528.7 | 287.5 | 493.0 | 335.0 | 654.0 | 7.3 |
| VII | 1 | 317.4 | 185.5 | 300.0 | 182.0 | 421.0 | 35.9 | 315.2 | 181.8 | 291.0 | 182.5 | 421.0 | 34.1 |
|  | 2 | 349.5 | 201.2 | 318.5 | 197.5 | 456.8 | 25.9 | 351.7 | 208.3 | 320.0 | 198.0 | 454.5 | 27.9 |
|  | 3 | 394.4 | 229.5 | 368.0 | 244.0 | 491.0 | 20.0 | 371.4 | 213.2 | 348.0 | 236.5 | 472.5 | 22.8 |
|  | 4 | 428.5 | 252.4 | 387.0 | 279.0 | 524.5 | 19.3 | 413.5 | 217.3 | 379.0 | 270.0 | 534.5 | 23.8 |
|  | 5 | 435.5 | 229.6 | 421.0 | 287.0 | 548.5 | 18.3 | 426.8 | 229.0 | 408.0 | 279.5 | 535.0 | 19.0 |
|  | 6 | 437.8 | 231.2 | 421.5 | 283.5 | 568.5 | 15.2 | 437.1 | 233.2 | 420.0 | 277.0 | 562.8 | 19.0 |
|  | 7 | 473.3 | 263.9 | 436.0 | 292.0 | 625.0 | 11.4 | 464.1 | 278.6 | 415.0 | 284.0 | 595.0 | 16.2 |
|  | 8 | 472.4 | 248.2 | 447.0 | 296.0 | 628.8 | 8.6 | 464.8 | 274.4 | 428.0 | 284.0 | 610.0 | 15.5 |
|  | 9 | 531.3 | 277.8 | 488.0 | 348.0 | 683.0 | 8.3 | 485.0 | 251.4 | 458.5 | 322.8 | 638.0 | 14.1 |
|  | 10 | 505.0 | 248.9 | 479.0 | 326.0 | 639.0 | 5.5 | 517.1 | 267.3 | 498.0 | 315.0 | 697.0 | 11.7 |
| VIII | 1 | 374.0 | 229.9 | 338.0 | 213.5 | 489.5 | 31.8 | 402.4 | 241.7 | 369.5 | 231.3 | 533.8 | 27.2 |
|  | 2 | 390.0 | 224.7 | 365.0 | 225.0 | 507.5 | 16.2 | 428.9 | 242.4 | 397.0 | 251.0 | 564.0 | 11.9 |
|  | 3 | 412.2 | 228.5 | 382.0 | 240.5 | 541.0 | 11.0 | 466.4 | 258.1 | 439.0 | 281.3 | 600.0 | 8.2 |
|  | 4 | 436.3 | 244.1 | 407.0 | 258.5 | 564.5 | 8.9 | 482.7 | 257.4 | 459.0 | 300.8 | 620.3 | 6.6 |
|  | 5 | 459.3 | 253.6 | 425.0 | 284.0 | 597.0 | 7.5 | 509.8 | 268.2 | 471.0 | 323.0 | 651.0 | 5.9 |
|  | 6 | 479.0 | 254.9 | 438.0 | 300.0 | 613.0 | 6.6 | 534.0 | 274.9 | 515.0 | 340.5 | 684.0 | 5.0 |
|  | 7 | 491.6 | 248.6 | 456.0 | 310.0 | 643.0 | 6.5 | 548.6 | 264.9 | 524.0 | 356.0 | 717.0 | 4.6 |
|  | 8 | 506.0 | 252.6 | 469.5 | 320.0 | 654.8 | 5.9 | 555.4 | 271.9 | 532.0 | 368.0 | 717.0 | 3.9 |
|  | 9 | 519.1 | 263.4 | 492.5 | 331.3 | 665.0 | 4.4 | 570.8 | 278.4 | 539.5 | 366.3 | 741.5 | 3.3 |
|  | 10 | 532.3 | 267.5 | 495.0 | 332.5 | 695.0 | 4.5 | 582.3 | 279.4 | 570.0 | 372.0 | 747.0 | 3.0 |
| IX | 1 | 326.1 | 176.9 | 316.0 | 194.5 | 419.0 | 23.1 | 339.7 | 177.1 | 327.5 | 208.3 | 437.0 | 19.3 |
|  | 2 | 364.9 | 190.3 | 345.5 | 229.2 | 466.7 | 13.1 | 366.6 | 190.7 | 350.5 | 230.3 | 463.0 | 16.3 |
|  | 3 | 400.2 | 214.3 | 374.5 | 250.0 | 508.0 | 10.8 | 397.5 | 203.7 | 384.0 | 248.0 | 512.0 | 9.8 |
|  | 4 | 431.9 | 240.2 | 402.0 | 279.0 | 530.0 | 8.7 | 423.6 | 207.0 | 416.0 | 267.8 | 525.0 | 8.7 |
|  | 5 | 450.1 | 221.9 | 420.5 | 293.2 | 574.7 | 7.3 | 436.8 | 211.1 | 421.0 | 283.0 | 558.5 | 7.5 |
|  | 6 | 472.3 | 232.6 | 441.0 | 303.0 | 604.5 | 6.7 | 448.7 | 208.9 | 423.5 | 300.0 | 579.8 | 5.6 |
|  | 7 | 485.9 | 241.3 | 462.5 | 302.0 | 605.8 | 5.2 | 464.7 | 220.7 | 458.0 | 307.8 | 597.0 | 6.0 |
|  | 8 | 498.6 | 250.8 | 471.5 | 310.5 | 626.5 | 4.0 | 475.5 | 221.7 | 479.0 | 312.8 | 613.8 | 4.8 |
|  | 9 | 509.3 | 241.2 | 484.5 | 335.7 | 662.2 | 4.4 | 483.3 | 223.8 | 473.5 | 323.0 | 623.0 | 4.6 |
|  | 10 | 515.2 | 244.9 | 501.0 | 351.0 | 678.0 | 3.9 | 506.3 | 238.3 | 489.0 | 327.5 | 625.5 | 4.4 |
| X | 1 | 366.2 | 201.1 | 350.0 | 226.0 | 482.0 | 28.1 | 380.9 | 213.6 | 366.0 | 227.8 | 502.5 | 24.4 |
|  | 2 | 404.5 | 215.9 | 388.5 | 241.0 | 530.3 | 13.4 | 413.7 | 221.7 | 400.0 | 246.5 | 550.8 | 16.8 |
|  | 3 | 437.6 | 234.2 | 422.0 | 261.5 | 561.0 | 12.2 | 443.6 | 241.5 | 417.0 | 260.0 | 578.5 | 12.1 |
|  | 4 | 463.6 | 241.0 | 435.0 | 286.5 | 604.0 | 12.1 | 464.6 | 243.9 | 439.5 | 291.0 | 606.0 | 12.1 |
|  | 5 | 488.4 | 260.1 | 457.0 | 313.0 | 630.0 | 12.0 | 483.6 | 252.3 | 450.0 | 299.8 | 637.3 | 12.1 |
|  | 6 | 493.7 | 251.0 | 472.0 | 319.0 | 624.5 | 9.5 | 493.3 | 248.1 | 477.0 | 320.5 | 641.5 | 11.6 |
|  | 7 | 507.1 | 256.4 | 492.0 | 337.0 | 651.0 | 9.2 | 503.7 | 253.5 | 493.5 | 320.0 | 652.0 | 9.3 |
|  | 8 | 512.7 | 266.4 | 485.0 | 327.5 | 652.5 | 8.5 | 515.9 | 254.4 | 498.0 | 325.3 | 665.8 | 9.3 |
|  | 9 | 525.2 | 263.8 | 507.0 | 347.0 | 664.0 | 7.4 | 524.6 | 265.9 | 509.0 | 344.5 | 673.0 | 8.9 |
|  | 10 | 543.4 | 278.2 | 520.5 | 347.0 | 699.5 | 6.2 | 524.2 | 282.5 | 508.0 | 324.5 | 668.5 | 8.5 |
| XI | 1 | 308.4 | 193.5 | 275.0 | 170.0 | 402.0 | 26.7 | 359.8 | 213.2 | 332.0 | 203.0 | 477.0 | 17.8 |
|  | 2 | 349.8 | 201.8 | 323.0 | 195.0 | 462.0 | 17.2 | 386.0 | 218.7 | 350.0 | 227.5 | 514.0 | 16.8 |
|  | 3 | 384.6 | 220.0 | 347.0 | 229.0 | 507.0 | 17.0 | 412.4 | 231.5 | 380.5 | 250.0 | 546.8 | 14.8 |
|  | 4 | 409.8 | 231.1 | 381.0 | 243.0 | 533.0 | 16.2 | 432.2 | 247.7 | 398.0 | 254.3 | 579.0 | 14.4 |
|  | 5 | 422.1 | 231.6 | 407.0 | 254.5 | 552.5 | 14.2 | 452.7 | 255.3 | 428.0 | 269.0 | 602.8 | 12.8 |
|  | 6 | 436.5 | 242.3 | 401.0 | 263.0 | 585.0 | 14.4 | 468.2 | 263.3 | 433.5 | 277.8 | 622.3 | 10.6 |
|  | 7 | 462.8 | 265.0 | 428.0 | 287.5 | 582.0 | 11.5 | 485.6 | 280.6 | 450.0 | 282.3 | 632.5 | 9.2 |
|  | 8 | 479.6 | 261.4 | 439.0 | 296.0 | 630.5 | 11.2 | 506.7 | 279.1 | 455.0 | 305.0 | 679.0 | 9.2 |
|  | 9 | 493.1 | 255.0 | 457.0 | 308.0 | 643.0 | 9.9 | 522.6 | 280.7 | 489.0 | 322.5 | 674.0 | 9.0 |
|  | 10 | 520.0 | 282.7 | 481.0 | 306.5 | 653.5 | 8.1 | 539.9 | 307.2 | 503.0 | 313.5 | 691.0 | 7.9 |
| XII | 1 | 292.1 | 177.8 | 266.0 | 157.0 | 391.0 | 32.1 | 303.9 | 181.1 | 272.5 | 170.8 | 407.8 | 33.3 |
|  | 2 | 331.7 | 191.7 | 304.5 | 195.0 | 456.0 | 22.9 | 323.2 | 181.3 | 297.0 | 196.5 | 436.5 | 24.3 |
|  | 3 | 367.0 | 210.7 | 319.5 | 209.3 | 507.8 | 23.9 | 366.2 | 209.2 | 323.0 | 203.5 | 494.0 | 20.3 |
|  | 4 | 386.3 | 219.2 | 354.0 | 225.0 | 513.0 | 20.9 | 383.8 | 213.3 | 353.0 | 227.5 | 502.5 | 18.9 |
|  | 5 | 404.1 | 210.2 | 385.0 | 255.5 | 517.5 | 20.4 | 400.4 | 220.5 | 360.5 | 242.3 | 528.3 | 18.6 |
|  | 6 | 421.9 | 226.1 | 395.0 | 263.0 | 561.0 | 19.6 | 416.1 | 246.9 | 375.0 | 237.5 | 545.5 | 17.4 |
|  | 7 | 437.9 | 229.0 | 430.0 | 280.0 | 572.0 | 20.6 | 427.9 | 245.1 | 401.0 | 244.5 | 565.5 | 16.2 |
|  | 8 | 457.5 | 250.0 | 435.0 | 268.0 | 590.5 | 20.8 | 426.3 | 230.4 | 409.5 | 249.8 | 562.0 | 15.2 |
|  | 9 | 468.8 | 244.8 | 459.0 | 293.0 | 608.5 | 19.1 | 443.7 | 242.8 | 422.0 | 275.0 | 575.0 | 13.5 |
|  | 10 | 477.7 | 260.3 | 460.0 | 283.3 | 628.3 | 18.0 | 460.3 | 236.5 | 450.0 | 299.0 | 593.0 | 12.5 |
| XIII | 1 | 335.0 | 180.7 | 320.0 | 213.0 | 427.8 | 20.9 | 373.4 | 202.5 | 362.0 | 236.5 | 477.0 | 17.4 |
|  | 2 | 372.5 | 191.4 | 357.0 | 234.3 | 476.8 | 13.1 | 406.3 | 205.8 | 392.0 | 260.0 | 518.0 | 13.9 |
|  | 3 | 401.1 | 193.3 | 388.5 | 266.0 | 512.8 | 12.0 | 436.5 | 227.8 | 417.0 | 280.0 | 561.0 | 10.3 |
|  | 4 | 431.1 | 210.9 | 400.0 | 286.0 | 549.0 | 10.6 | 457.7 | 237.3 | 432.0 | 295.0 | 581.0 | 10.1 |
|  | 5 | 455.3 | 220.8 | 443.5 | 296.0 | 589.8 | 9.6 | 478.8 | 241.1 | 460.0 | 311.0 | 618.0 | 8.4 |
|  | 6 | 483.9 | 236.8 | 466.0 | 327.0 | 612.8 | 8.8 | 485.4 | 240.5 | 471.0 | 321.0 | 644.5 | 7.9 |
|  | 7 | 503.5 | 244.1 | 477.0 | 334.0 | 645.0 | 8.7 | 497.4 | 256.0 | 483.0 | 317.0 | 657.0 | 7.6 |
|  | 8 | 518.7 | 243.7 | 503.0 | 359.8 | 665.3 | 6.8 | 516.8 | 271.2 | 498.0 | 329.0 | 670.0 | 6.9 |
|  | 9 | 544.5 | 260.2 | 524.5 | 365.3 | 671.0 | 5.9 | 534.5 | 275.8 | 506.5 | 336.8 | 708.8 | 6.5 |
|  | 10 | 573.9 | 274.6 | 543.5 | 403.5 | 733.3 | 4.8 | 548.4 | 289.9 | 516.0 | 357.3 | 709.3 | 6.1 |

I - Manaus – FMT; II - Belém UREDIP; III - Santarém - Municipal STF; IV - Recife - HC/UFPE ; V – Jab. dos Guararapes - MUNICIPAL STF; VI - Salvador – HUPES; VII - Salvador – CEDAP; VIII - Rio de Janeiro – IPEC; IX Belo Horizonte – UFMG; X - São Paulo - CRT/SP; XI - SAE S.J.R. Preto - MUNICIPAL STF; XII - São Paulo - Municipal Network; XIII - Porto Alegre - PARTENON

NDA - No data available

HIV/BVS - HIV Cohort Brazilian Study

SISCEL - Laboratory Tests Control System

SD – Standard Deviation

VL – Viral Load

Det - (%) - Above

Med – Mean

M - Median
